# Beyond ion homeostasis: WNK1 and the role of water influx in T cell biology

**DOI:** 10.3389/fimmu.2026.1995432

**Published:** 2026-10-30

**Authors:** Robert Köchl, Victor L. J. Tybulewicz

**Affiliations:** 1 Peter Gorer Department for Immunobiology, King’s College London, London, United Kingdom; 2 The Francis Crick Institute, London, United Kingdom

**Keywords:** T cell activation, T cell development, T cell migration, water flux, WNK1

## Abstract

The serine-threonine kinase WNK1 is a regulator of ion homeostasis and cell volume control studied most extensively in the kidney. WNK1 phosphorylates and activates the OXSR1 and STK39 kinases, which in turn phosphorylate members of the SLC12A-family of ion co-transporters resulting in influx of Na^+^, K^+^ and Cl^-^ ions and subsequent osmotically-driven water entry through aquaporins and an increase in cell volume. This WNK1-regulated pathway restores cell volume following shrinkage caused by exposure to hyperosmotic conditions, a process termed regulatory volume increase (RVI). Unexpectedly, we discovered that WNK1 regulates multiple aspects of T cell biology. During thymic development, WNK1-deficient thymocytes fail to progress beyond the β-selection checkpoint. In mature CD4^+^ T cells, WNK1 is required for T cell migration and homing to secondary lymphoid organs. During T cell activation, WNK1 is required for proliferation. Mechanistically, we showed that T cell antigen receptor (TCR) and chemokine receptor signalling in CD4^+^ T cells cause activation of WNK1 resulting in ion and water influx. Furthermore, this WNK1-induced water influx is required for chemokine-induced migration and for entry into cell cycle during T cell activation, illustrating the importance of water uptake in T cell biology. However, it remains unclear how WNK1 is activated downstream of the TCR and chemokine receptors, and how water entry affects intracellular signalling pathways. In this review, we summarise the current knowledge of WNK1 signalling in T cells and discuss how WNK1 may be activated, and whether localised changes in water content might modulate spatially-restricted signalling events in T cells.

## 1 Introduction

CD4^+^ and CD8^+^ T cells develop in the thymus and once mature, emigrate through the blood and populate secondary lymphoid organs (SLOs) such as lymph nodes and spleen. These mature naive T cells continuously migrate through SLOs in search of cognate antigen. If they locate antigen, they form stable immune synapses with antigen-presenting cells (APCs), undergo activation, growth and proliferation, and subsequently migrate into other tissues where they orchestrate immune responses and eliminate infected or transformed cells (1–4). These functions require rapid changes in transcription, cytoskeletal organisation, adhesion and migration. Much research has focused on how these processes are regulated by signalling pathways downstream of the T cell antigen receptor (TCR), chemokine and cytokine receptors. However, comparatively little is known about the biophysical constraints that T cells experience and how ion transporters and channels, as well as osmotic processes, regulate their function.

An unexpected insight into this came from our unbiased RNAi screen for kinases regulating integrin-dependent adhesion in Jurkat T cells. The screen identified the serine/threonine kinase WNK1 as a negative regulator of integrin-mediated adhesion, showing that WNK1-deficient T cells had hyperactive LFA-1-mediated adhesion (5). This was surprising because WNK1 had previously been studied almost exclusively in epithelial and renal physiology, where it plays a central role in salt transport and blood pressure regulation (6, 7). However, at the time, there were no studies of WNK1 in lymphocytes, or other immune cells. Our subsequent work showed that WNK1 regulates thymocyte development, T cell migration, activation and proliferation (5, 8–10) and performs similar roles in B cells (11).

## 2 WNK1 activation and regulation of cell volume

WNK1 belongs to a family of serine/threonine kinases that includes four catalytically active members, WNK1-4, and the related pseudokinases NRBP1 and NRBP2 (12, 13). The family is characterised by an unusual kinase domain in which the catalytic lysine residue in the ATP-binding site is located outside the canonical position found in most protein kinases, giving rise to the name “With-No-Lysine” (WNK) kinases (14, 15).

Hyperosmotic conditions cause cell shrinkage which triggers activation of WNK1. Activated WNK1 phosphorylates the related OXSR1 and STK39 kinases (also known as OSR1 and SPAK), which in turn phosphorylate and regulate the activity of several members of the SLC12A-family of cation-coupled chloride co-transporters (16). Phosphorylation by OXSR1 and STK39 activates the SLC12A1 (NKCC2) and SLC12A2 (NKCC1) ion co-transporters, which mediate the coupled influx of Na^+^, K^+^ and 2Cl⁻ ions into the cell (17, 18). At the same time, phosphorylation of the SLC12A4 (KCC1), SLC12A5 (KCC2), SLC12A6 (KCC3) and SLC12A7 (KCC4) co-transporters inhibits the efflux of K^+^ and Cl⁻ from the cell (16). Together, these changes lead to a net accumulation of intracellular ions. The resulting osmotic gradient drives water influx through aquaporins, causing the cell to swell. This process, termed regulatory volume increase (RVI), restores cell volume following hyperosmotically-driven shrinkage and typically occurs within minutes (19).

Several mechanisms have been proposed for how WNK1 is activated. Chloride ions directly bind to the catalytic site of the kinase domain of WNK1 and inhibit its autophosphorylation and activation (20). Structural studies have shown that chloride ions promote the formation of an inactive WNK1 dimer (21, 22). Reduction of the Cl^-^ concentration induces de-dimerisation of WNK1 and its activation. In addition, in response to hypertonic conditions, WNK1 rapidly moves into condensates, seen as puncta in imaging experiments (23). These biomolecular condensates contain active WNK1 along with WNK1 substrates such as OXSR1 and STK39, which, presumably, is where they are phosphorylated by WNK1. WNK1 condensation depends on its large intrinsically disordered region (IDR), whose removal blocks both condensation and activation. Thus, condensation is likely essential for WNK1 activation. It was proposed that formation of WNK1 condensates is driven by macromolecular crowding that occurs following hypertonicity-induced cell shrinkage (23). However, a more recent study showed that formation of protein condensates can be driven by the reduced water potential that results from hypertonicity-induced water efflux (24). Whether changes in water potential drive WNK1 condensation remains unknown.

Our work has shown that in CD4^+^ T cells, WNK1 is also activated by signalling from the TCR and the CCR7 chemokine receptor (5). Pharmacological inhibition of phosphoinositide 3-kinase (PI3K) or AKT abolishes this activation, placing these kinases upstream of WNK1 in these signalling pathways (5). However, neither PI3K nor AKT appears to directly activate WNK1. While AKT can phosphorylate WNK1, this does not activate WNK1 (25). Thus, the molecular mechanism linking receptor-proximal PI3K and AKT signalling to WNK1 activation remains unknown.

The WNK1–OXSR1–STK39 pathway regulates ion transport and osmotically-driven water influx in several cell types. Given the central role of this pathway in controlling cell volume and hydration, it is likely that WNK1-dependent water influx also operates in T cells.

## 3 WNK1 in thymocyte development

T cell development occurs in the thymus and involves several checkpoints that eliminate T cells expressing TCRs that are non-functional or recognise self. At the first such checkpoint, termed β-selection, CD4^-^CD8^-^ double negative (DN) thymocytes rearrange TCRβ genes. Successful rearrangement results in expression of TCRβ protein which binds pre-Tα to form the pre-TCR (26). Signalling from the pre-TCR promotes thymocyte survival, extensive proliferation and differentiation from DN to double positive (DP, CD4^+^CD8^+^) thymocytes. Subsequently, DP thymocytes rearrange TCRα genes, and express TCRα protein forming a TCRαβ receptor. The cells then undergo positive and negative selection to select for cells expressing a TCR which can bind to self-peptide MHC complexes with low but not high affinity, becoming either CD4^+^CD8^-^ or CD4^-^ CD8^+^ single positive thymocytes which then emigrate into the periphery as CD4^+^ or CD8^+^ naive T cells (27).

Deletion of *Wnk1* using CD2-Cre, which is expressed at the DN thymocyte stage, led to an almost complete developmental block at the β-selection checkpoint, resulting in very few naive T cells being produced (10). WNK1-deficient DN thymocytes had greatly reduced pre-TCR-induced proliferation and differentiation into DP thymocytes. Similarly, mice in which OXSR1 and STK39 signalling is disrupted, as well as mice lacking the ion co-transporter SLC12A2, display a similar albeit weaker phenotype, suggesting that the canonical WNK1–OXSR1–STK39–SLC12A2 pathway is required for progression beyond the β-selection checkpoint (10). Mechanistically, loss of WNK1 resulted in decreased expression of MYC-regulated genes, including genes required for entry into the cell cycle (10). Consistent with this, retroviral overexpression of MYC in WNK1-deficient thymocytes partially rescued the developmental defect (10).

There is no direct evidence that WNK1-dependent changes in ion transport or water influx are required during thymocyte development. However, disruption of OXSR1 and STK39 signalling or loss of SLC12A2 results in similar, albeit milder, developmental defects, suggesting that ion and water influx may indeed contribute to β-selection. However, the progressively weaker phenotypes seen when these downstream WNK1 pathway components are disrupted indicates that WNK1 regulates additional pathways independent of OXSR1, STK39 and SLC12A2, which may not involve ion and water transport.

## 4 WNK1 in T cell migration

After leaving the thymus, naive T cells continuously circulate between blood and SLOs, searching for cognate antigen on APCs. Entry into lymph nodes occurs via high endothelial venules and depends on a coordinated regulation of adhesion to the endothelium via LFA-1–ICAM-1 interactions and transmigration in response to the CCL19 and CCL21 chemokines signalling through CCR7 (28, 29).

We found that phosphorylated (active) WNK1, phosphorylated OXSR1 and phosphorylated SLC12A2 localise to the leading edge of CD4^+^ T cells migrating in response to CCL21, suggesting a role in controlling migration (9). Consistent with this, deletion of *Wnk1* using a tamoxifen-inducible Cre recombinase impaired homing of naive CD4^+^ T cells to lymph nodes *in vivo* and strongly reduced migration on ICAM-1-coated surfaces in response to CCL21 *in vitro*, while integrin-mediated adhesion was increased (5, 9). Loss of WNK1 in activated CD4^+^ T cells also resulted in impaired migration. However, the increased adhesion of WNK1-deficient T cells did not underly the reduced migration as integrin blocking *in vivo*, did not restore interstitial migration speed (5). WNK1-deficient CD4^+^ T cells were smaller and failed to increase in size upon chemokine stimulation, unlike wild-type cells (9). Moreover, they showed decreased cellular polarisation and F-actin polymerisation in response to CCL21 (9). Decreased migration was also observed in CD4^+^ T cells with disrupted OXSR1 and STK39 signalling, as well as in SLC12A2-deficient cells, although these defects were weaker than those seen in the absence of WNK1, again implying that WNK1 transduces some CCR7 signals independently of OXSR1, STK39 and SLC12A2 (5, 9).

Mechanistically, these data suggest that during CCL21-induced migration, WNK1 acts in part through OXSR1 and STK39 and SLC12A-family ion transporters to induce ion influx and subsequent water entry across the plasma membrane (5, 9). In agreement with this, treatment of CD4^+^ T cells with CCL21 results in a WNK1-dependent increase in K^+^ ions, water entry and cell volume (9). Remarkably, migration of WNK1-inhibited CD4^+^ T cells could be partially rescued by treating them with hypoosmotic medium, which forces water into the cells, demonstrating that water influx and/or the resulting volume increase are required for efficient migration (9). Since activation of the WNK1 pathway is polarised to the leading edge, WNK1-induced ion and water influx may also occur at the leading edge. This localised water entry likely increases the spacing between the plasma membrane and the F-actin cortex, facilitating actin polymerisation, membrane protrusion and directional migration (9). Consistent with this, in WNK1-inhibited cells spacing is reduced between the F-actin cortex and the plasma membrane at the leading edge, suggesting that insufficient local water uptake may limit the physical space available for actin filament extension (**Figure 1A**) (9). Taken together, these findings identify the WNK1 pathway as a key regulator of T cell migration, coupling chemokine receptor signalling to localised ion and water influx at the leading edge and subsequent actin polymerisation.

**Figure 1:**
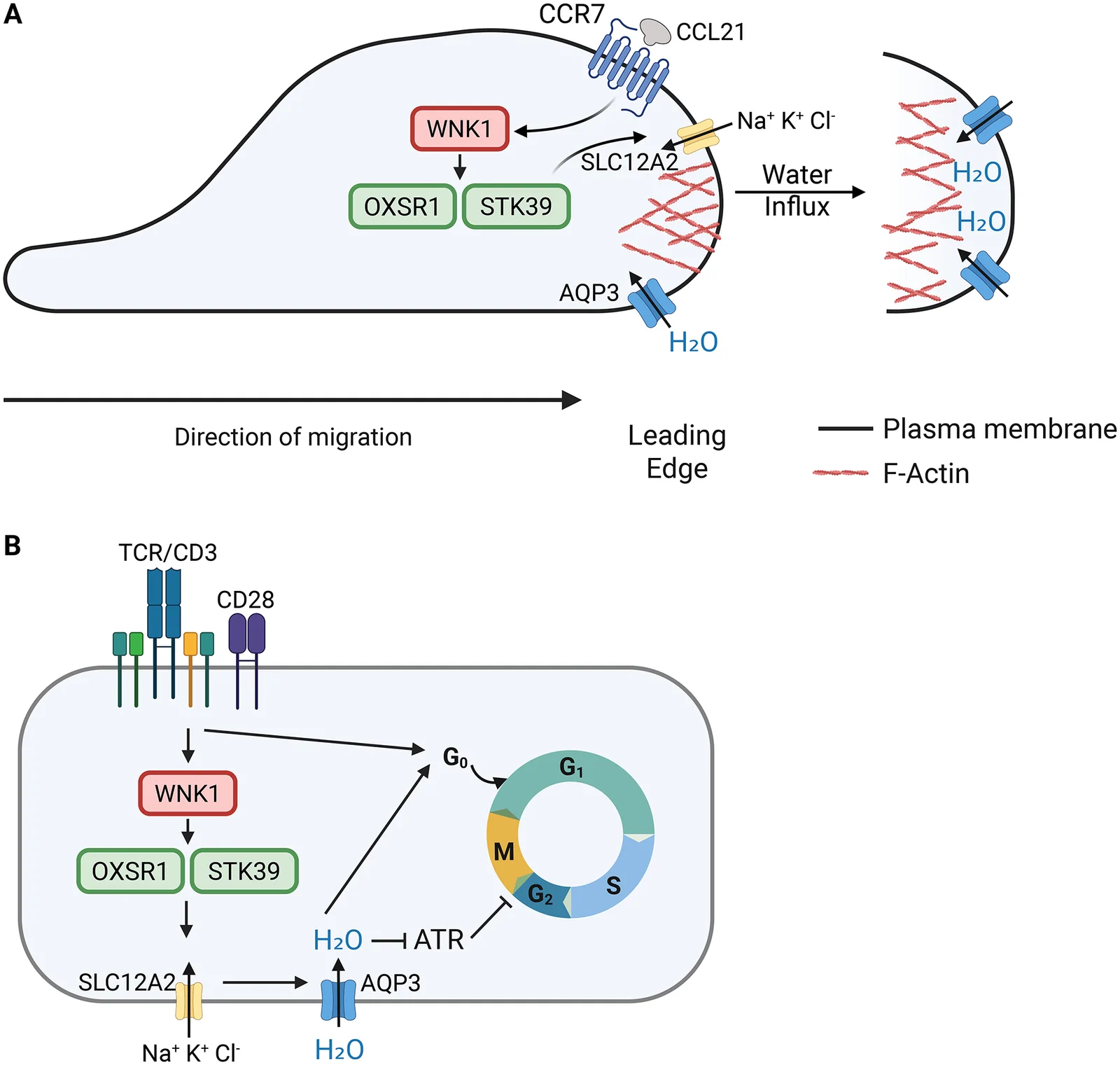
WNK1-regulated water influx is required for T cell migration and proliferation. **(A)**. Binding of CCL21 to CCR7 results in activation of WNK1, phosphorylation and activation of OXSR1 and STK39 and SLC12A-family ion co-transporters, resulting in Na^+^, K^+^ and Cl^-^ influx followed by water entry via aquaporins such as AQP3, all localised at the leading edge of the migrating T cell. Water is required to swell the plasma membrane away from the underlying F-actin generating space into which new F-actin filaments can extend thereby pushing the membrane forward (9). **(B)**. Stimulation of CD4^+^ T cells through TCR and CD28 causes activation of WNK1, OXSR1, STK39 and SLC12A-family ion co-transporters resulting in an ion influx which causes water entry. Water is required for entry into cell cycle (G0 to G1 transition). WNK1 is also required for DNA synthesis during S phase and for transition of the G2/M checkpoint by inhibiting ATR activity (8). Figures created in Biorender.

## 5 WNK1 in CD4^+^ T cell activation and proliferation

Following recognition of cognate antigen presented on APCs, naive T cells stop migrating and form stable immune synapses that can persist for several hours (30, 31). Sustained TCR signalling induces a rapid growth phase, followed by proliferation and differentiation into effector T cells (4). During this process, T cells downregulate the lymph node homing receptor CCR7 and upregulate tissue-homing receptors that enable migration into peripheral tissues (32, 33).

We found that stimulation of CD4^+^ T cells through the TCR and the CD28 co-stimulatory receptor using anti-CD3ε and anti-CD28 antibodies rapidly activates WNK1 (5). Genetic ablation of WNK1 increased T cell–APC conjugate formation (5) and caused a strong block in TCR-CD28-induced proliferation, which was also observed following disruption of OXSR1 and STK39 signalling (8). *In vivo*, WNK1 is also essential for the generation of T follicular helper (Tfh) cells and a class-switched antibody response following NP-CGG immunisation (8). Increased integrin-dependent adhesion, however, is unlikely to explain the *in vitro* proliferation defect, as WNK1-deficient T cells respond poorly when stimulated with anti-CD3ε and anti-CD28 in the absence of ICAM-1. Nevertheless, a contribution of hyperactive integrins to the defective activation *in vivo*, cannot be excluded. Indeed, mice with a constitutively active LFA-1 integrin, show impaired T cell responses (34).

Further analysis showed that WNK1 is required for TCR-CD28-induced activation of ERK kinases, and WNK1, OXSR1 and STK39 are required for entry into the cell cycle, efficient DNA synthesis in S phase and suppression of the ATR-mediated G2/M cell cycle checkpoint (8). Again, we showed that TCR-CD28 stimulation results in a WNK1-dependent increase in the rate of K^+^ ion influx and a volume increase consistent with water influx. Notably, incubating WNK1-inhibited CD4^+^ T cells in hypoosmotic media to force water into the cells rescued defects in TCR-CD28-induced ERK activation and cell cycle entry, demonstrating that WNK1-dependent ion fluxes and subsequent osmotically-driven water influx in response to TCR signalling are required for T cell proliferation (**Figure 1B**) (8).

## 6 Future directions

These findings establish WNK1 as a critical regulator of thymocyte development and CD4^+^ T cell migration, activation and proliferation. A unifying theme is that WNK1-dependent ion and water influxes underlie these processes (**Figures 1A, B**). However, several questions remain unresolved, and the full scope of WNK1 signalling in T cells is likely broader than the canonical WNK1–OXSR1–STK39–SLC12A pathway.

### 6.1 How is WNK1 activated in T cells

In non-immune cells, hyperosmotic stress and the resulting macromolecular crowding or decreased water potential drive WNK1, OXSR1 and STK39 into biomolecular condensates (23, 24). Formation of these condensates is proposed to concentrate the pathway and drive its activation, providing a mechanism by which WNK1 directly senses changes in cellular hydration (23, 24). Once activated, WNK1 signalling through OXSR1, STK39 and the SLC12A-family of ion co-transporters causes ion and water influx, restoring cell volume and dissolving the condensates (23). In addition, chloride ions bind directly to the WNK1 kinase domain and inhibit autophosphorylation through formation of an inactive WNK1 dimer, suggesting that reductions in intracellular chloride may additionally promote kinase activation, although the physiological relevance of this mechanism remains to be established (20–22).

In CD4^+^ T cells, engagement of CCR7 and the TCR by their respective ligands leads to WNK1 activation which is dependent on PI3K and AKT signalling (5). However, it is not known how PI3K and AKT activity leads to WNK1 activation. To date, no upstream kinase, other than WNK trans-phosphorylation (35), has been shown to directly activate WNK1 in any cell type, and the nature of the connection between receptor-proximal signalling and WNK1 activation loop phosphorylation is an important unresolved question.

An interesting hypothesis is that signalling from CCR7 and TCR results in a reduction in water potential which would cause WNK1 to move into condensates, become active and phosphorylate co-condensed OXSR1 and STK39. Moreover, since phosphorylated WNK1, phosphorylated OXSR1 and phosphorylated SLC12A2 localise to the leading edge of migrating T cells (9), if WNK1 is being activated in these locations by condensation, it would imply that there may be local reductions in water potential driven by CCR7 or TCR signalling. In this model, since WNK1 activation leads to water influx, the kinase would function as both a sensor and effector of local changes in cellular hydration and water potential. To address this hypothesis, it will be important to determine if receptor-proximal signalling can trigger the formation of either cell-wide or localised WNK1 condensates, and, if so, whether these are caused by receptor-induced changes in water potential. Such a finding would raise the question of how CCR7 and TCR signalling reduces water potential.

### 6.2 How does water influx influence T cell function

Treatment with hypoosmotic media rescues some phenotypes in WNK1-deficient T cells demonstrating that water entry itself is critical for multiple aspects of T cell biology. Rather than acting as a classical biochemical second messenger, changes in cellular water content may represent a physical regulator of signalling that functions by modulating water potential, molecular crowding, diffusion or mechanical properties of membranes.

In naive CD4^+^ T cells, WNK1 deficiency leads to both impaired migration and reduced actin polymerisation in response to CCL21 (9). Hypoosmotic treatment rescued migration, consistent with water influx at the leading edge causing increased spacing between the plasma membrane and the underlying F-actin network, thereby allowing actin filaments to polymerise into this space and push the membrane forward (9). However, a direct link between water influx and actin polymerisation has not been formally established.

The requirement for water influx in cell migration is not unique to T cells. Ion influx and aquaporin-mediated water entry at the leading edge has been shown to contribute to migration in other cell types as well, including endothelial tip cells, cancer cells and neutrophils, both through actin-dependent and -independent mechanisms (36–39). Notably, S1P-induced bleb-based T cell motility requires WNK1-dependent water influx in addition to myosin contractility, suggesting that water-driven migration may operate across multiple modes of T cell movement (40).

Water influx may also alter the biochemical environment in which signalling takes place, by reducing cytoplasmic macromolecular crowding or increasing water potential. Cell volume increases in other cell types have been shown to alter protein concentrations and consequently affect binding equilibria and reaction kinetics (41, 42). Conversely, cell shrinkage induced by hyperosmotic treatment slows intracellular signalling as a result of increased crowding (43), and decreases water potential which promotes the formation of biomolecular condensates thereby enhancing or reorganising signalling pathways (24). This effect of water influx on macromolecular crowding or water potential may explain why WNK1-regulated water entry is required for TCR-induced ERK activation and Ki67 expression (8). However, whether water influx also contributes to the WNK1-dependent regulation of integrin-mediated adhesion is unknown. Taken together, water influx likely influences T cell function by altering the biochemical and physical environment in which signalling takes place.

### 6.3 Are there WNK1 signalling outputs beyond the canonical OXSR1–STK39–SLC12A axis

Disruption of OXSR1 and STK39 or SLC12A2 resulted in weaker T cell phenotypes compared to loss or inhibition of WNK1, implying that WNK1 induces pathways beyond the canonical OXSR1-STK39-SLC12A pathway leading to ion influx (5, 8–10). WNK1 has also been implicated in signalling processes in non-immune cells (44), including mTORC2–SGK1 signalling, AXL-regulated metastasis and autophagy (45–47). However, whether these are truly independent of WNK1-regulated ion and water influx remains to be explored.

### 6.4 WNK1 in biophysically altered tissues and cancer

T cells infiltrating peripheral tissues, and particularly tumours, encounter environments that can biophysically differ from the lymphoid organs in which they develop and are activated. These environments are characterised by elevated osmolarity, altered ionic composition, elevated interstitial pressure, increased mechanical stiffness and confinement (48). This raises the possibility that WNK1 signalling in tissue-infiltrating T cells is regulated not only by receptor signalling, but also directly by the biophysical properties of the environment. In a hyperosmotic tumour environment, T cell shrinkage could activate WNK1 and induce an RVI response. Similarly, physical confinement during migration may alter T cell volume and water content, potentially engaging WNK1-dependent volume regulation in response. Whether osmotic or mechanical properties of tissues regulate WNK1 activity and T cell function remains unknown.

WNK1 is also expressed in cancer cells where it can promote invasion and metastasis, potentially making it an attractive oncological target (47). However, given the requirement for WNK1 for T cell migration and homing, systemic WNK1 inhibition may also impair T cell recruitment into tumours and thereby suppress anti-tumour immunity.

## 7 Concluding remarks

WNK1 controls ion flux and water influx at multiple stages of T cell development and function and this activity is required for T cells to undergo the rapid changes in cell morphology, volume and cytoskeletal organisation that underlie migration and activation.

## References

[1] Chopp L Redmond C O'Shea JJ Schwartz DM . From thymus to tissues and tumors: A review of T-cell biology. J Allergy Clin Immunol. (2023) 151:81–97. doi: 10.1016/j.jaci.2022.10.011 36272581 PMC9825672

[2] Schenkel JM Pauken KE . Localization, tissue biology and T cell state - Implications for cancer immunotherapy. Nat Rev Immunol. (2023) 23:807–23. doi: 10.1038/s41577-023-00884-8 37253877 PMC11448857

[3] Ashby KM Hogquist KA . A guide to thymic selection of T cells. Nat Rev Immunol. (2024) 24:103–17. doi: 10.1038/s41577-023-00911-8 37464188

[4] Sinclair LV Cantrell DA . Protein synthesis and metabolism in T cells. Annu Rev Immunol. (2025) 43:343–66. doi: 10.1146/annurev-immunol-082323-035253 40279310

[5] Kochl R Thelen F Vanes L Brazao TF Fountain K Xie J . Wnk1 kinase balances T cell adhesion versus migration *in vivo*. Nat Immunol. (2016) 17:1075–83. doi: 10.1038/ni.3495 27400149 PMC4994873

[6] Rodan AR Jenny A . Wnk kinases in development and disease. Curr Top Dev Biol. (2017) 123:1–47. doi: 10.1016/bs.ctdb.2016.08.004 28236964 PMC5329892

[7] Richardson C Alessi DR . The regulation of salt transport and blood pressure by the Wnk-Spak/Osr1 signalling pathway. J Cell Sci. (2008) 121:3293–304. doi: 10.1242/jcs.029223 18843116

[8] Biggs O'May J Vanes L de Boer LL Lewis DA Hartweger H Kunzelmann S . Wnk1-dependent water influx is required for Cd4+ T cell activation and T cell-dependent antibody responses. Nat Commun. (2025) 16:1857. doi: 10.1038/s41467-025-56778-x 39984435 PMC11845700

[9] de Boer LL Vanes L Melgrati S Biggs O'May J Hayward D Driscoll PC . T cell migration requires ion and water influx to regulate actin polymerization. Nat Commun. (2023) 14:7844. doi: 10.1038/s41467-023-43423-8 38057317 PMC10700356

[10] Kochl R Vanes L Llorian Sopena M Chakravarty P Hartweger H Fountain K . Critical role of Wnk1 in Myc-dependent early mouse thymocyte development. Elife. (2020) 9:e56934. doi: 10.7554/eLife.56934 33051000 PMC7591260

[11] Hayward DA Vanes L Wissmann S Sivapatham S Hartweger H Biggs O'May J . B cell-intrinsic requirement for Wnk1 kinase in antibody responses in mice. J Exp Med. (2023) 220:e20211827. doi: 10.1084/jem.20211827 36662229 PMC9872328

[12] Shekarabi M Zhang J Khanna AR Ellison DH Delpire E Kahle KT . Wnk kinase signaling in ion homeostasis and human disease. Cell Metab. (2017) 25:285–99. doi: 10.1016/j.cmet.2017.01.007 28178566

[13] Magana-Avila G Carbajal-Contreras H Amnekar RV Dite T Tellez-Sutterlin M Garcia-Avila K . Nrbp1 and Tsc22d proteins affect distal convoluted tubule physiology through modulation of the Wnk pathway. Sci Adv. (2025) 11:eadv2083. doi: 10.1126/sciadv.adv2083 40668923 PMC12266109

[14] Xu BE Lee BH Min X Lenertz L Heise CJ Stippec S . Wnk1: Analysis of protein kinase structure, downstream targets, and potential roles in hypertension. Cell Res. (2005) 15:6–10. doi: 10.1038/sj.cr.7290256 15686619

[15] Min X Lee BH Cobb MH Goldsmith EJ . Crystal structure of the kinase domain of Wnk1, a kinase that causes a hereditary form of hypertension. Structure. (2004) 12:1303–11. doi: 10.1016/j.str.2004.04.014 15242606

[16] Alessi DR Zhang J Khanna A Hochdorfer T Shang Y Kahle KT . The Wnk-Spak/Osr1 pathway: Master regulator of cation-chloride cotransporters. Sci Signal. (2014) 7:re3. doi: 10.1126/scisignal.2005365 25028718

[17] Vitari AC Thastrup J Rafiqi FH Deak M Morrice NA Karlsson HK . Functional interactions of the Spak/Osr1 kinases with their upstream activator Wnk1 and downstream substrate Nkcc1. Biochem J. (2006) 397:223–31. doi: 10.1042/BJ20060220 16669787 PMC1479760

[18] Richardson C Sakamoto K de los Heros P Deak M Campbell DG Prescott AR . Regulation of the Nkcc2 ion cotransporter by Spak-Osr1-dependent and -independent pathways. J Cell Sci. (2011) 124:789–800. doi: 10.1242/jcs.077230 21321328 PMC3114804

[19] Hoffmann EK Lambert IH Pedersen SF . Physiology of cell volume regulation in vertebrates. Physiol Rev. (2009) 89:193–277. doi: 10.1152/physrev.00037.2007 19126758

[20] Piala AT Moon TM Akella R He H Cobb MH Goldsmith EJ . Chloride sensing by Wnk1 involves inhibition of autophosphorylation. Sci Signal. (2014) 7:ra41. doi: 10.1126/scisignal.2005050 24803536 PMC4123527

[21] Teixeira LR Akella R Humphreys JM He H Goldsmith EJ . Water and chloride as allosteric inhibitors in Wnk kinase osmosensing. Elife. (2024) 12:RP88224. doi: 10.7554/eLife.88224 39584807 PMC11588334

[22] Akella R Humphreys JM Sekulski K He H Durbacz M Chakravarthy S . Osmosensing by wnk kinases. Mol Biol Cell. (2021) 32:1614–23. doi: 10.1091/mbc.E20-01-0089 33689398 PMC8684725

[23] Boyd-Shiwarski CR Shiwarski DJ Griffiths SE Beacham RT Norrell L Morrison DE . Wnk kinases sense molecular crowding and rescue cell volume via phase separation. Cell. (2022) 185:4488–506. doi: 10.1016/j.cell.2022.09.042 36318922 PMC9699283

[24] Watson JL Seinkmane E Styles CT Mihut A Kruger LK McNally KE . Macromolecular condensation buffers intracellular water potential. Nature. (2023) 623:842–52. doi: 10.1038/s41586-023-06626-z 37853127 PMC10665201

[25] Vitari AC Deak M Collins BJ Morrice N Prescott AR Phelan A . Wnk1, the kinase mutated in an inherited high-blood-pressure syndrome, is a novel Pkb (protein kinase B)/Akt substrate. Biochem J. (2004) 378:257–68. doi: 10.1042/BJ20031692 14611643 PMC1223938

[26] von Boehmer H . Unique features of the pre-T-cell receptor alpha-chain: Not just a surrogate. Nat Rev Immunol. (2005) 5:571–7. doi: 10.1038/nri1636 15999096

[27] Klein L Kyewski B Allen PM Hogquist KA . Positive and negative selection of the T cell repertoire: What thymocytes see (and don't see). Nat Rev Immunol. (2014) 14:377–91. doi: 10.1038/nri3667 24830344 PMC4757912

[28] von Andrian UH Mempel TR . Homing and cellular traffic in lymph nodes. Nat Rev Immunol. (2003) 3:867–78. doi: 10.1038/nri1222 14668803

[29] Girard JP Moussion C Forster R . Hevs, lymphatics and homeostatic immune cell trafficking in lymph nodes. Nat Rev Immunol. (2012) 12:762–73. doi: 10.1038/nri3298 23018291

[30] Mempel TR Henrickson SE Von Andrian UH . T-cell priming by dendritic cells in lymph nodes occurs in three distinct phases. Nature. (2004) 427:154–9. doi: 10.1038/nature02238 14712275

[31] Dustin ML . The immunological synapse. Cancer Immunol Res. (2014) 2:1023–33. doi: 10.1158/2326-6066.CIR-14-0161 25367977 PMC4692051

[32] Zhu J Yamane H Paul WE . Differentiation of effector Cd4 T cell populations (*). Annu Rev Immunol. (2010) 28:445–89. doi: 10.1146/annurev-immunol-030409-101212 20192806 PMC3502616

[33] Kaech SM Cui W . Transcriptional control of effector and memory Cd8+ T cell differentiation. Nat Rev Immunol. (2012) 12:749–61. doi: 10.1038/nri3307 23080391 PMC4137483

[34] Semmrich M Smith A Feterowski C Beer S Engelhardt B Busch DH . Importance of integrin Lfa-1 deactivation for the generation of immune responses. J Exp Med. (2005) 201:1987–98. doi: 10.1084/jem.20041850 15955836 PMC2212031

[35] Thastrup JO Rafiqi FH Vitari AC Pozo-Guisado E Deak M Mehellou Y . Spak/Osr1 regulate Nkcc1 and Wnk activity: Analysis of Wnk isoform interactions and activation by T-loop trans-autophosphorylation. Biochem J. (2012) 441:325–37. doi: 10.1042/BJ20111879 22032326 PMC3242505

[36] Nagy TL Strickland E Weiner OD . Neutrophils actively swell to potentiate rapid migration. Elife. (2024) 12:RP90551. doi: 10.7554/eLife.90551 38953885 PMC11219036

[37] Stroka KM Jiang H Chen SH Tong Z Wirtz D Sun SX . Water permeation drives tumor cell migration in confined microenvironments. Cell. (2014) 157:611–23. doi: 10.1016/j.cell.2014.02.052 24726433 PMC4365996

[38] Zhang Y Li Y Thompson KN Stoletov K Yuan Q Bera K . Polarized Nhe1 and Swell1 regulate migration direction, efficiency and metastasis. Nat Commun. (2022) 13:6128. doi: 10.1038/s41467-022-33683-1 36253369 PMC9576788

[39] Kondrychyn I He L Wint H Betsholtz C Phng LK . Combined forces of hydrostatic pressure and actin polymerization drive endothelial tip cell migration and sprouting angiogenesis. Elife. (2025) 13:RP98612. doi: 10.7554/eLife.98612 39977018 PMC11841990

[40] Rodriguez FS De Belly H Zhang Y Strickland E Frazer GL Ojukwu J . S1p induces bleb-based T cell motility via S1pr1-dependent activation of Rhoa and Wnk1. bioRxiv. (2025) 10.03.680076. doi: 10.1101/2025.10.03.680076 41256389 PMC12622039

[41] Minton AP . The influence of macromolecular crowding and macromolecular confinement on biochemical reactions in physiological media. J Biol Chem. (2001) 276:10577–80. doi: 10.1074/jbc.R100005200 11279227

[42] Delarue M Brittingham GP Pfeffer S Surovtsev IV Pinglay S Kennedy KJ . Mtorc1 controls phase separation and the biophysical properties of the cytoplasm by tuning crowding. Cell. (2018) 174:338–49. doi: 10.1016/j.cell.2018.05.042 29937223 PMC10080728

[43] Miermont A Waharte F Hu S McClean MN Bottani S Leon S . Severe osmotic compression triggers a slowdown of intracellular signaling, which can be explained by molecular crowding. Proc Natl Acad Sci USA. (2013) 110:5725–30. doi: 10.1073/pnas.1215367110 23493557 PMC3619343

[44] McCormick JA Ellison DH . The Wnks: Atypical protein kinases with pleiotropic actions. Physiol Rev. (2011) 91:177–219. doi: 10.1152/physrev.00017.2010 21248166 PMC3035565

[45] Saha B Dellova DCAL Demko J Sørensen MV Takagi E Gleason CE . Wnk1 is a chloride-stimulated scaffold that regulates Mtorc2-activity and ion transport. J Cell Sci. (2022) 135(23):jcs260313. doi: 10.1242/jcs.260313 36373794 PMC9789407

[46] Gallolu Kankanamalage S Lee AY Wichaidit C Lorente-Rodriguez A Shah AM Stippec S . Wnk1 is an unexpected autophagy inhibitor. Autophagy. (2017) 13:969–70. doi: 10.1080/15548627.2017.1286431 28282258 PMC5446055

[47] Jaykumar AB Jung JU Parida PK Dang TT Wichaidit C Kannangara AR . Wnk1 enhances migration and invasion in breast cancer models. Mol Cancer Ther. (2021) 20:1800–8. doi: 10.1158/1535-7163.MCT-21-0174 34253593 PMC9013269

[48] Mohammadi H Sahai E . Mechanisms and impact of altered tumour mechanics. Nat Cell Biol. (2018) 20:766–74. doi: 10.1038/s41556-018-0131-2 29950570

